# Comparing farmers’ perception of climate effect on cocoa yield with climate data in the Humid zone of Nigeria

**DOI:** 10.1016/j.heliyon.2023.e23155

**Published:** 2023-12-03

**Authors:** Joseph O. Adejuwon, Kehinde E. Tewogbade, Olusegun Oguntoke, Gideon C. Ufoegbune

**Affiliations:** a Department of Water Resources Management and Agrometeorology, Federal University of Agriculture, P.M.B. 2240, Abeokuta, Ogun State, Nigeria; b Department of Environmental Management and Toxicology, Federal University of Agriculture, P.M.B. 2240, Abeokuta, Ogun State, Nigeria

**Keywords:** Cocoa yield, Humid environment, Meteorological parameters, Tropics, Africa

## Abstract

Apart from the conventional inputs for cocoa production, climatic parameters have a significant effect on the yield of the crop globally. However, existing literature suffers a knowledge gap on the farmers' perceptions of climate effect on cocoa yield and its nexus with climate data. The chi-square test of independence, standardized anomaly index (SAI), multiple regression, z-distribution, and descriptive statistics were employed to investigate the socioeconomic and climate impacts on yield. The registered farmers (280) from the Osun State Cocoa Growers Association (OSCGA) in the Nigerian Humid Zone were the interviewees. Climate data were retrieved from the records of the Nigerian Meteorological Agency, Abuja, while the cocoa yield data were provided by the Ministry of Agriculture, Osogbo from 1999 to 2019. The results revealed that climate affected cocoa yield and the yield was significant at p ≤ 0.05 in 1999 and 2000. Most farmers perceived that low temperature, relative humidity, rainfall, sunshine, and wind speed influenced cocoa yield positively. Farmers’ perception was influenced by age, marital status, household size, and educational level at p ≤ 0.01. Understanding the climate and its consequences on cocoa yield will help develop management practices, that were hitherto lacking in Osun State, Nigeria. This study presents the socioeconomic and climate effects on cocoa yield and makes recommendations for management practices in regions with similar ecological settings.

## 1 Introduction

Cocoa is one of the major tree crops in West Africa. African cocoa production was over 72 % of the world's total, with about 90 % of it produced in West Africa from 2013 to 2017 [1,2]. South America and Asia are also major cocoa producers. The reason for cocoa's prominence could be attributed to its importance as a commercial crop, foreign exchange earner, and its level of consumption [3]. Recently, cocoa production in Nigeria has experienced significant stagnation. Nigeria dropped from the third-largest producer of cocoa in 2013/2014 with a production of 248,000 metric tons to the sixth position in 2016/2017 with a production of 230,000 metric tons [1,2]. The smallness of farm holdings, inadequate roads, depleting farm labour, low capital investment, and unfavourable climatic change were the prominent factors identified for the stagnation [4]. Other factors include poor soil quality and unfavourable climatic factors, especially rainfall and temperature [5,6]**.** The perception that farmers have about climate change informs their planting decisions and determines the adoption of adaptation measures [7].

Cocoa was one of the major cash and export crops in Nigeria before crude oil came into prominence in 1958. The country ranked the second-largest producer of cocoa globally by 1965 due to the rapid utilization of the crop [8]. Cocoa is largely produced in southwestern Nigeria and part of eastern Nigeria. The top cocoa-producing states in Nigeria are Ondo, Cross River, Ogun, Akwa Ibom, Edo, Ekiti, Delta, Osun, and Oyo state [9]. Ondo State is the largest producer of cocoa in Nigeria and accounts for over 40 % of all cocoa exports [10,11].

Studies have indicated that crop production and yield were affected by climate, soil fertility, insect and pest infestations, planting period, and agronomic practices including land preparation, choice of cultivars, planting density, fertilizers, application of irrigation water, pesticides, and herbicides [12,13]. However, some of these factors are either directly or indirectly influenced by climate. Climate is very important to agricultural production in the humid ecological area of Nigeria where farmers depend largely on rainfall. A change in climate influences cocoa production by changing the reproduction of pests and diseases and altering the host's physiology and resistance [14,15]. The extended drought period causes newly transplanted young cocoa plants and some cocoa trees to wither, while major pests and diseases of cocoa are promoted by favourable climatic situations. The black pod disease is a core danger to cocoa production when the relative humidity is very high [16]. Kurukulasuriya et al. [17] and Deressa et al. [18] revealed that climate change impacted Africa's agriculture negatively and that adaptation is one of the policy options for reducing the negative impact of climate change.

Few studies existed on farmers' perception of climate on crops [[19], [20], [21]], but none were reported for cocoa. Also, the research on the effect of climate on cocoa production was scanty [5,6,22,23], with none on yield. However, the current literature suffers a knowledge gap on the effect of climate on cocoa yield concerning farmers' perception, and its nexus with climate data. This study aimed at comparing climate data with the farmers’ opinions about climate parameters concerning cocoa yield, which could affect their response to advocated adaptation measures. The objectives of the study are to.

i. assess the socioeconomic characteristics of cocoa farmers and their perception of the weather's influence on cocoa yield.
ii. determine the climate (annual rainfall, temperature, relative humidity, sunshine, and wind speed) departure from normal in the study area
iii. examine the influence of meteorological parameters on cocoa yield in the study area
iv. analyze the magnitude of variability of crop yield and assess the significance of their normal year-by-year distribution

## 2 Materials and method

### 2.1 Study area

**Fig. 1a:**
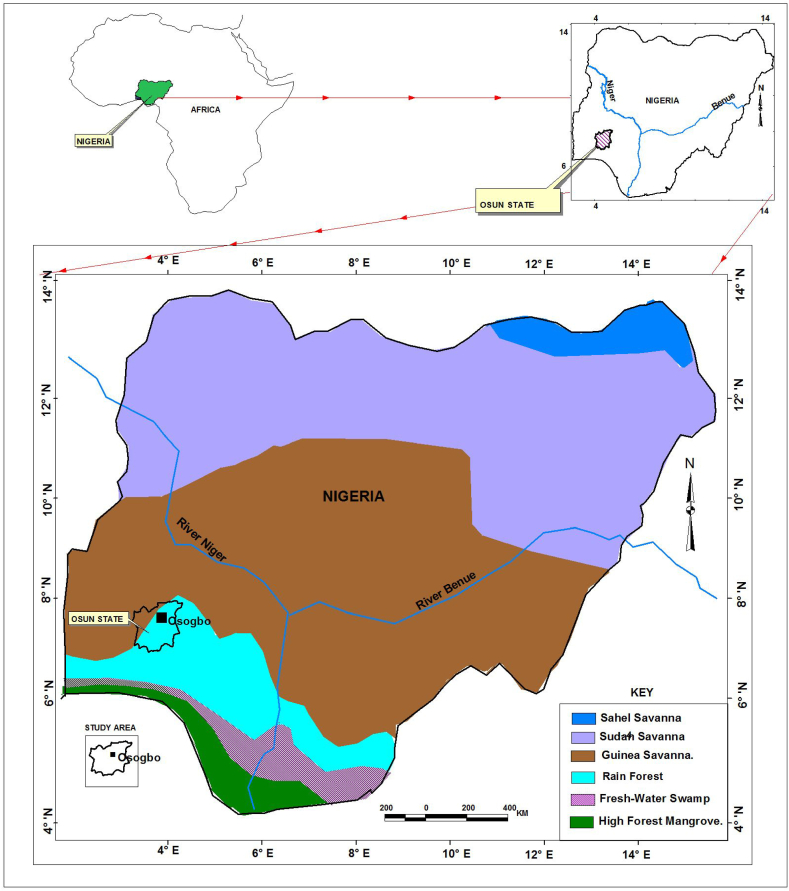

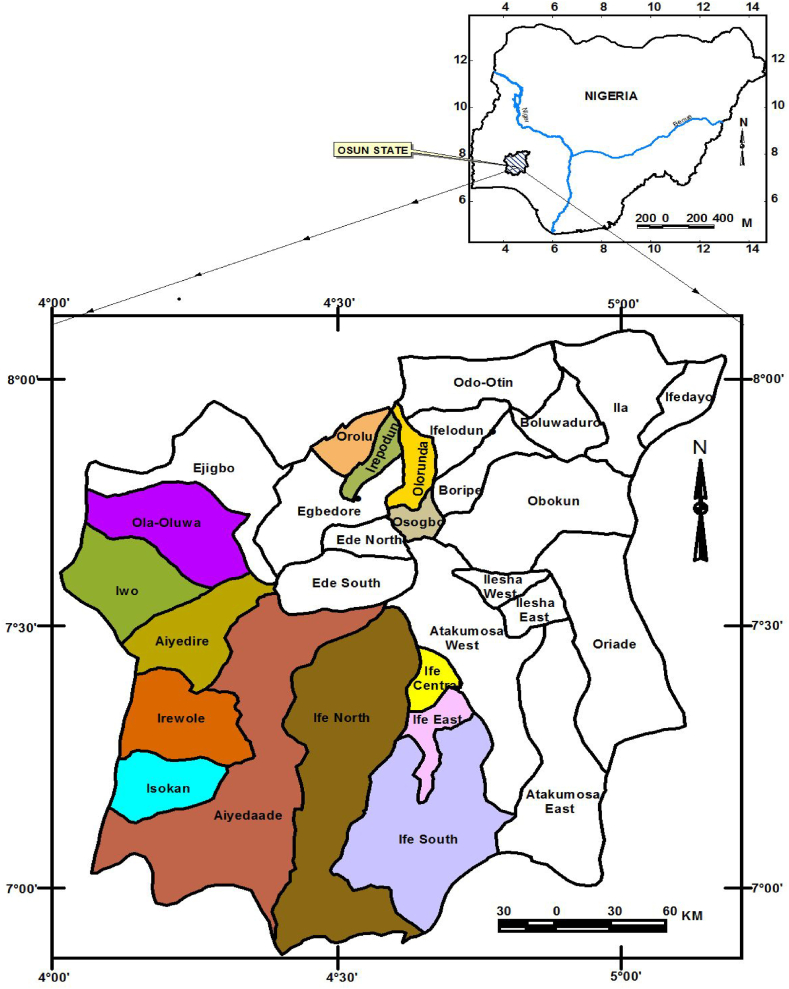
Ecological zones of Nigeria and study area. Fig. 1b: Local Government Area selected for questionnaire administration.

Osun State, Nigeria lies between longitude 04°E and 06°E and latitude 06°N and 09°N (Fig. 1a and b). The study area is in the rainforest ecological zone. The state is bounded by Ogun State to the south, Kwara State to the North, Oyo State to the west, and Ekiti and Ondo to the east. Osun State is one of the top cocoa-producing states in Nigeria [9]. The State experiences distinct dry and wet seasons annually. The dry season is controlled by tropical continental (cT) air mass, which originates from the Sahara Desert while the wet season is controlled by tropical maritime (mT) air mass from the Atlantic Ocean [[24], [25], [26], [27], [28], [29], [30], [31], [32], [33]]. The dry season is from November to February while the wet season lasts from March to October. The mean annual rainfall is 1360.3 mm while the mean annual temperature is 31.3 °C. The area encounters a short dry season in the mid-wet season of July/August months [[34], [35], [36], [37]].

### 2.2 Data

#### 2.2.1 Data collection

##### 2.2.1.1 Field investigation

The primary data were obtained through a field investigation, involving direct interactions with the cocoa farmers (respondents). A questionnaire was used to elicit information on the socioeconomic characteristics of the farmers, and their perception of the influence of climate parameters on cocoa yield. A multi-stage sampling technique was adopted to select 20 farmers from each of the 14 towns in the three agricultural zones (Ife, Osogbo, and Iwo) in Osun state. The first stage was the purposive selection of Iwo, Ife, and Osogbo local government areas

of Osun state due to the large number of cocoa farmers in the three local government areas. Purposive sampling, also known as judgmental, selective, or subjective sampling, is a form of.

non-probability sampling in which researchers rely on their judgment when choosing members of the population to participate in their surveys. The second stage was the selection of the headquarters and major towns of these local government areas: Iwo zone (Iwo, Ileogbo, Bode-Osi, Gbongan, Ikire, Apomu), Ife zone (Ajebamide, Oke-Ogbo, Ipetumodu, Ifetedo), and Osogbo zone (Igbona, Ilobu, Ifon-Osun). The third stage was a simple random selection of 20 farmers from each of the selected towns. The interviewed farmers were registered farmers with

the Osun State Cocoa Growers Association (OSCGA). In all, a total of 280 cocoa farmers were interviewed.

##### 2.2.1.2 Secondary data

Climate data (rainfall, temperature, humidity, sunshine, and wind speed) was retrieved from the archive of the Nigerian Meteorological Agency (NIMET; Abuja), while the cocoa yield data was sourced from the Ministry of Agriculture, Osogbo from 1999 to 2019. The climate data was collected for Osogbo station because it is the only synoptic station in Osun State. The study was limited to this period due to the unavailability of cocoa yield records in the state until 1999.

#### 2.2.2 Data analysis

Data were analyzed using descriptive statistics (means, frequency counts, percentages), the chi-square test of independence, standardized anomaly index (SAI), multiple regression, and z-distribution. The chi-square test of independence was employed in this study to test for a significant relationship between the farmers' socioeconomic data and the perception of cocoa yield as well as the significant relationship between the farmers’ socioeconomic data and their perception of the influence of weather parameters on cocoa yield. This statistical method is appropriate for the study and preferred to other methods because it can be used in studies dealing with demographics; and applied in a wide area including surveys, business decision-making, quality control, biological research, and medical research among others [38,39].

where x^2^ = Chi-square statistics, Σ = summation operator, O = observed frequency and E = Expected frequency.

The degree of freedom for chi-square is computed as DF = (R– 1) (C −1)

where DF = Degree of freedom, R = Row and C = Column.

The SAI was used to determine the annual rainfall, temperature, relative humidity, sunshine, and wind speed departure from normal (mean). The indices near zero indicate normal rainfall, while those substantially above or below zero indicate relatively wet or dry conditions [[40], [41], [42]]. SAI has the advantage of a provision of more information about the magnitude of the anomalies because influences of dispersion have been removed.

For each station i, annual rainfall/temperature/relative humidity/sunshine/wind speed ri is normalized to produce a series of annual departures as follows:

SAI = (r_i_ - r_ij_)/Ơ_*i*_

Where:

ri = the mean annual rainfall/temperature/relative humidity/sunshine/wind speed at station i over the entire length of the record.

rij = the annual rainfall/temperature/relative humidity/sunshine/wind speed for station i in the year j.

Ơ = the standard deviation of the annual rainfall/temperature/relative humidity/sunshine/wind speed at station i.

Multiple regression was employed to determine the influence of meteorological parameters on cocoa yield. The yield of cocoa is the dependent variable in the multiple regression analysis while rainfall, temperature, sunshine, wind speed, and relative humidity were the independent variables. Multiple regression was analyzed using Statistical Package for Social Sciences (SPSS Version 20). The coefficient of determination was calculated to examine the extent to which climate determined cocoa yield.

Z-distribution was used in this study to show the magnitude of variability of crop yield and

assess the significance of their normal year-by-year distribution. It is denoted as:

_

Z-distribution = X - X.

SD.Where:

X_i_ = Annual yield, $\bar{X}$ = Mean annual yield, SD = Standard deviation.

The Z-distribution values > −1.5 or 1.5 showed a significant impact at p ≤ 0.05 while values > - 2.0 or 2.0 indicated a significant impact at p ≤ 0.01 [43].

## 3 Results

### 3.1 Farmers’ perception of climate effect on cocoa yield

The result shows that 75.4 % of the cocoa farmers were males while 24.6 % were females (Table 1). The farmers were aged between 31 and 45 years (41.8 %) followed by 45–60 years (33.9 %) while the least were those between 15 and 30 years. Ninety-eight percent (97.9 %) of the respondents relied on cocoa farming as their primary occupation and were married. Only 43.2 % of the respondents had formal education while 56.8 % received non-formal education. The household size of the respondent varied from 1 member to more than 6 members.

**Table 1:** Socioeconomic characteristics of the respondents.

Variable	Option	Frequency	Percentage
Gender	Male	211	75.4
	Female	69	24.6
Age range (years)	15–30 years	16	5.7
	31–45 years	117	41.8
	45–60 years	95	33.9
	Above 60 years	52	18.6
Occupation	Farmer	274	97.9
	Civil servant	6	2.1
Household Size	1	5	1.8
	2–3	42	15.0
	4–6	120	42.9
	Above 6	113	40.4
Marital Status	Single	4	1.4
	Married	274	97.9
	Divorced	2	7
Have the respondents acquired formal education?	Yes	121	43.2
	No	159	56.8

**Note**: The total frequency of respondents for each variable is 280 (100 %).

The respondents who raised their cocoa through the nursery (where seeds are planted and raised into young plants before being transplanted to the field) were 93.9 % (Table 2). Ninety-two percent (91.5 %) of the farmers produce cocoa on a large scale; 95.7 % produce between 1 and 5 tons per annum. About 61 % and 38 % rated the yield of cocoa as good and excellent in the study area. The setbacks that confronted farmers concerning cocoa production included parasite infection (42.5 %), weed control (39.6 %), and unfavourable weather conditions (17.9 %). The proportion of farmers that received incentives (chemicals, seedlings, and loans) ranged from 20.4 to 49.3 %. In a bid to increase or improve cocoa production, 23.2, 29.3, and 47.5 % of farmers indicated the provision of chemicals for pest control, proper management of farms, and proper maintenance of farm inputs.

**Table 2:** Farmer's perception of cocoa yield and related factors.

Variable	Option	Frequency	Percentage
Cocoa yield	Excellent	105	37.5
	Good	170	60.7
	Fair	2	0.7
	Poor	3	1.1
Is cocoa yielding income for the three local government areas?	Yes	274	97.9
	No	6	2.1
Is cocoa produced on a large scale?	Yes	257	91.8
	No	23	8.2
Quantity produced per year	1–5 tons	263	95.7
	6–10 tons	6	2.1
	11–15 tons	1	4.0
	16–20 tons	5	1.8
Has the yield of cocoa reduced in recent years	Yes	150	53.6
	No	130	56.4
Preparatory method for cocoa cultivation	Nursery	263	93.9
	Others	17	6.1
The danger associated with cocoa cultivation	Parasite	119	42.5
	Weed	111	39.6
	Weather conditions	50	17.9
The effort of the government to increase cocoa yield	Loan	138	49.3
	Seedlings	85	30.4
	Chemicals	57	20.4
Ways in which cocoa production can be improved	Proper management	133	47.5
	Proper maintenance	82	29.3
	Appropriate use of chemicals	65	23.2

**Note**: Total frequency of respondents for each variable is 280 (100 %).

**Fig. 2:**
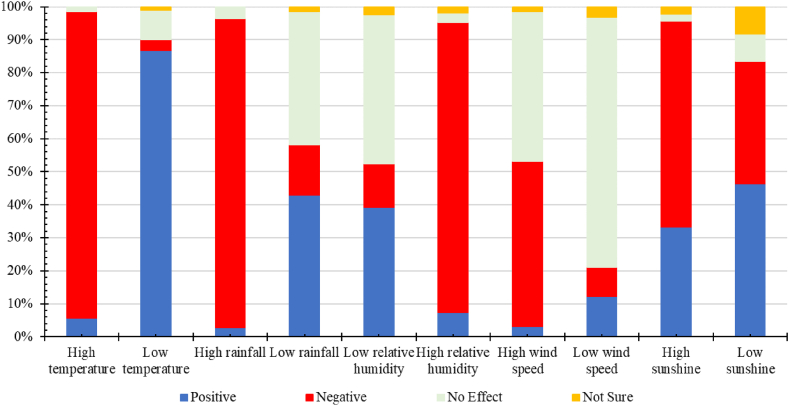
Farmer's perception of the influence of weather parameters on cocoa yield.

Eighty-seven percent (86.8 %) of the farmers perceived that low temperature influenced cocoa yield positively, and between 39.7 and 46.4 % indicated a positive association with low relative humidity, rainfall, or sunshine (Fig. 2). Over 75 % of the farmers indicated that low wind speed does not affect yield while 12.1 % believed wind speed had a positive effect. On the other hand, 87.9–93.6 % observed that high relative humidity, temperature, or rainfall.

influenced cocoa yield negatively. Similarly, 50 % and 65.5 % linked high wind speed and sunshine to the negative influence.

**Fig. 3:**
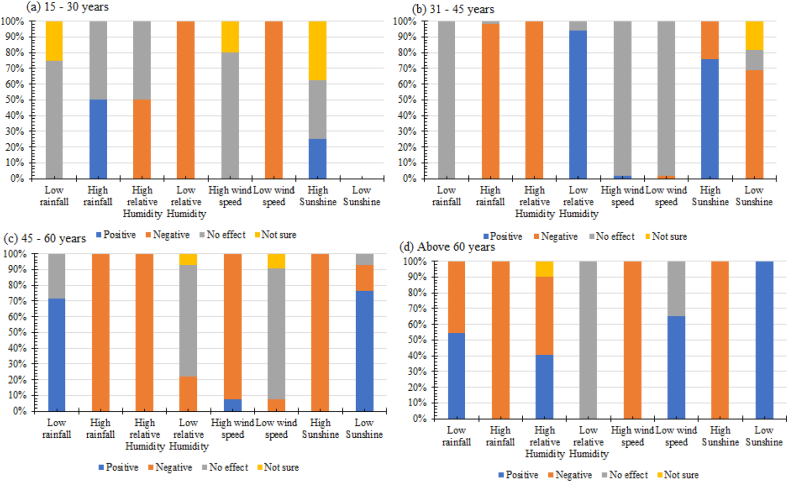
Farmers' age-group perception about the influence of weather parameters on cocoa yield.

**Fig. 4:**
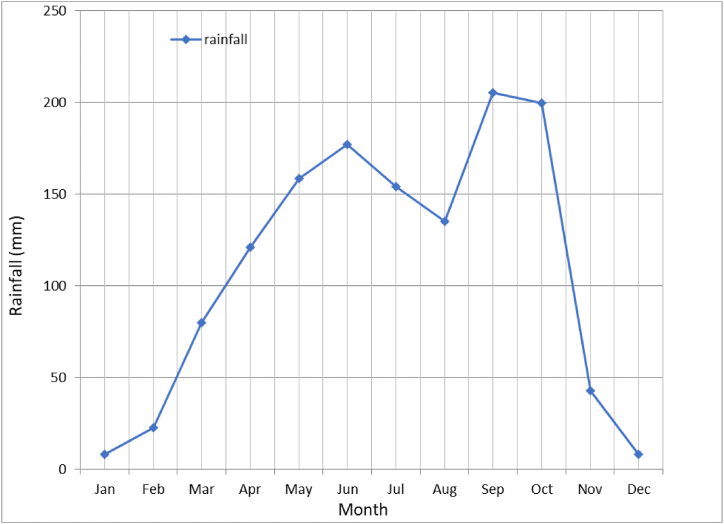
The mean monthly rainfall (mm) in Osogbo, Nigeria.

The most experienced farmers (above 60 years) who made up 18.6 % of the total farmers understood that high rainfall, temperature, wind speed, and sunshine harmed cocoa yield (Fig. 3). Besides, half of these farmers knew that high relative humidity hurt cocoa yield. All the farmers also noted that low temperature, rainfall, sunshine, and wind speed had a positive effect. For example, the mean monthly rainfall (mm) in Osun State, Nigeria showed that rainfall is high during the wet season (Fig. 4). However, 75–100 % of farmers aged 15–30 years, which represent 4.2–5.7 % of the entire farmer population, noted that high wind speed, low temperature, low rainfall, and low sunshine did not affect cocoa yield. Also, all the farmers believed that low relative humidity and low wind speed harmed yield.

**Fig. 5:**
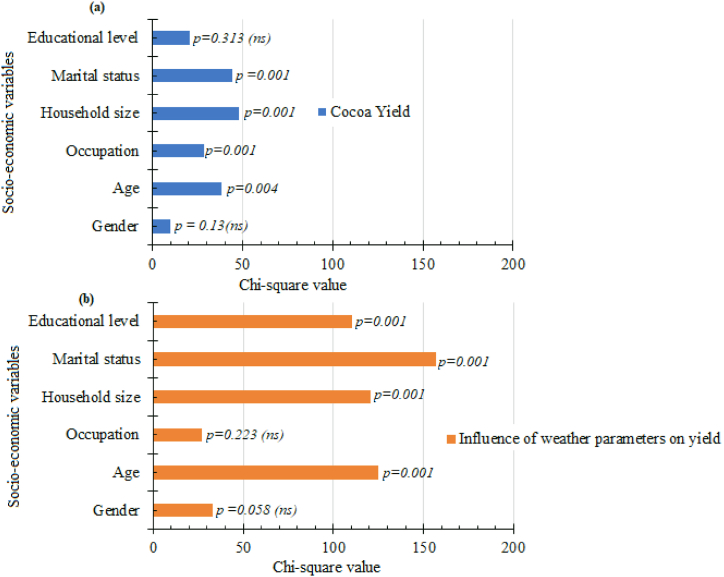
Relationship between the farmers' socioeconomic data perception on cocoa yield and the influence of weather parameters.

As shown in Fig. 5a, farmers' socio-economic variables of occupation, age, marital status, and household size were significantly (p ≤ 0.01) related to the perception of cocoa yield. The values ranged from 28.73 to 47.80. However, gender and educational level did not show a significant relationship with the farmers' perception of cocoa yield. Moreover, farmers' socioeconomic data (educational level, age, marital status, and household size) with a value ranging from 110.37 to 157.05 have a significant (p ≤ 0.01) influence on their perception of weather parameters on cocoa yield (Fig. 5b). Conversely, gender and occupation showed no significant relationship with the farmers’ perception of weather on cocoa yield.

### 3.2 Climate variability and cocoa yield

**Fig. 6:**
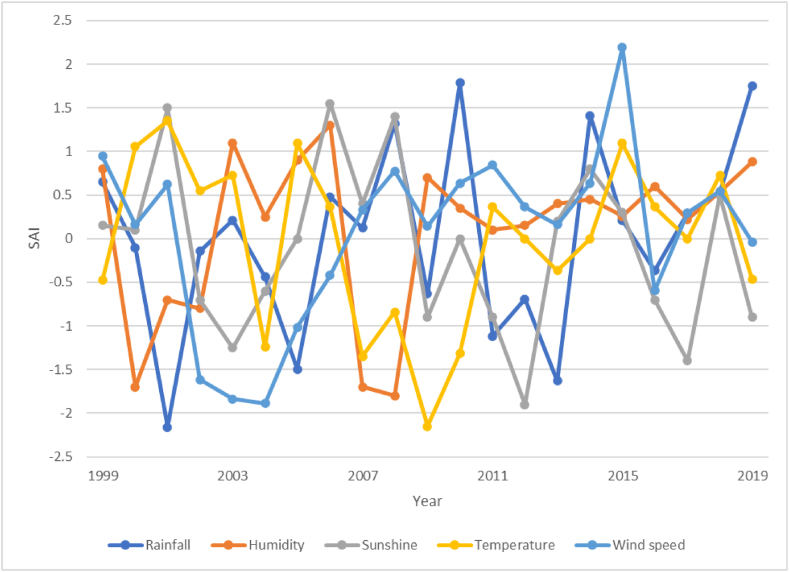
Standardized Rainfall, Humidity, Sunshine, Temperature, and Wind speed Anomaly index for Osogbo.

Fig. 6 shows the annual climatic parameters of rainfall, mean annual temperature, humidity, wind, and sunshine anomaly in Osun State. Rainfall was above average for about half of the years studied, severely high in 2010 and 2019, and lowest in 2001 when extreme drought.

occurred. The relative humidity was moderately above average in 2003 and 2006 but severely below average in 2000, 2007, and 2008. The sunshine was in excess for most of the study period.

However, it was severe in 2012. The wind speed reduced from moderate in 1999 to the lowest in 2004. The wind speed of 2002–2004 was very severe. Thereafter the windspeed was above average from 2007 except in 2016 and 2019 when it was below average. The wind speed was extremely high in 2015. Temperature anomaly was positive for most years, and negative for a few years with extreme in 2009.

The multiple regression prediction of the influence of meteorological parameters on cocoa yield in Osun State, Nigeria is shown in Table 3. The model shows that the weather parameters (sunshine hour, wind speed, relative humidity, temperature, and rainfall) do not have a significant influence on cocoa yield. The coefficient of determination revealed that 42 % of cocoa yield was determined by climate variability. The implication is that 58 % of the variance in

**Table 3:** Multiple regression model for cocoa yield in Osun State, Nigeria.

Station	Regression Model	R	R^2^	Significance
Osogbo	364044.45 + 1487.29SH-5518.19*WS-269.66RH- 2.269RF-8562.75T	0.705	0.417	0.246

*Significant α 0.05 and **α 0.01.

SH-sunshine hour, WS-wind speed, RH- relative humidity, RF- rainfall, T- Temperature.

cocoa yield is explainable by other factors than climatic elements considered.

**Fig. 7:**
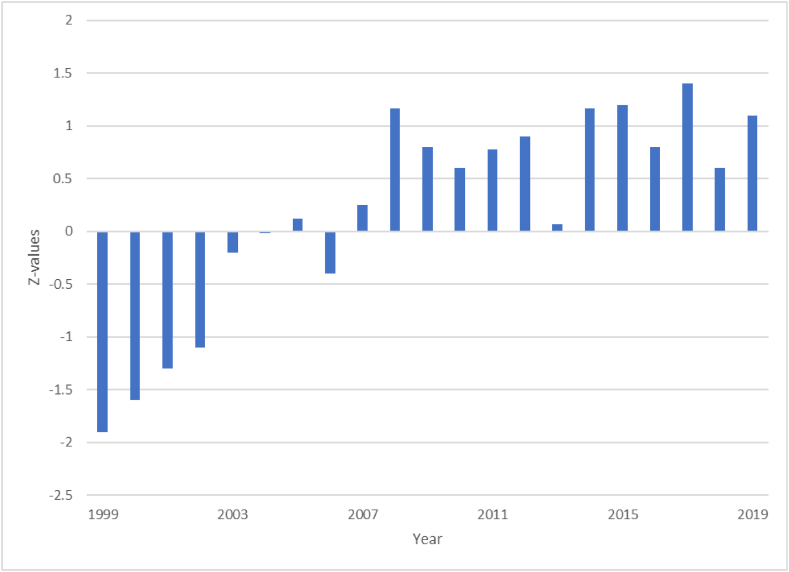
Cocoa yield anomalies in Osun State, Nigeria.

Fig. 7 shows the cocoa yield anomaly in the study area. The yield was negative from 1999 to 2006 and positive from 2007 to 2019. A significant yield was experienced at p ≤ 0.05 in 1999 and 2000. The positive yield indicates an increase in yield while the negative yield shows decreased yield. The two anomaly periods that were significant at a 95 % level of confidence during the investigation constituted 9.52 % of the anomalies while the remaining 19 anomalies (90.48 %) depicted ordinary exit from normal yield and did not impact so much on the farmers’ means of living. The cocoa yield was best in 2017 when climatic conditions appeared to be more favourable to farmers, and worst during the droughts of 1999 and 2000 when cocoa recorded the highest negative yield anomalies. The negative yield period was characterized by cocoa yield failure. Rainfall and humidity were less than the threshold values while temperature and sunshine.

were higher. For instance, the annual rainfall of 1367.9 mm in 2000 was less than the average rainfall of 1385 mm. The yield increased and was higher than the average when the weather conditions improved from 2007 onward.

## 4 Discussion

### 4.1 Socioeconomic characteristics and farmers’ perception of climate on cocoa yield

Cocoa farming is a male-dominated business in the study area. This may not be unrelated to the drudgery involved in farming and also, the fact that land ownership for farming activities is a paternal affair in the southwestern part of Nigeria [44]. The consideration of cocoa farming as a full-time occupation may explain why all the farmers were married. Marriage and family size play prominent roles in subsistence agriculture generally in Africa and particularly in Nigeria. Marital status determines the per capita food availability in the family [45]. Kiriti and Tisdell [45] show that the average per capita food availability in Kenyan shillings is highest for married women living with husbands followed by the married women living alone, and least among the female-headed households. This is because women living with husbands work longer hours than those married but living alone, and also longer than unmarried women [46]. Eremi et al. [47] reported that marital status was positive and significant at a 0.5 % confidence level, implying that as women get married, their propensity to engage in cocoa production increases more than that of single ladies. The rural women were involved in land preparation for cocoa plantations, promotion of nurseries, planting of seeds and seedlings, weeding, spraying of chemicals, gathering of cocoa pods, and transportation and storage. Similarly, since labour is required at weeding and harvesting times, wives and children become handy and reduce the number of hired labour required. Little wonder that most farmers have at least four members in their households. According to Akinnagbe and Ajayi [48], and Popoola et al. [49], a large household means the involvement of more hands in cocoa production. Davis et al. [50] reported a positive significant relationship at a 1% significance level between family size and subsistence farming production. They stated that household size sometimes determines the food consumption of the household which means that the larger the household size, the more the household is likely to produce its food through subsistence farming. This is an indication of enough farm labour through family labour, as well as increased food availability in the household. Akinnagbe et al. [51] studied the intra-household roles in cocoa production in Ondo State and discovered that family labour (81.4 %) was the main source of labour used in cocoa production and that men plant the cocoa seed at the nursery (90 %) while women were mostly involved in the assemblage of harvested pods (68 %). Men and women were involved in transplanting seedlings to permanent sites (84.3 %, 81.4 %) and removal of beans from the pod (72.2 %, 80.0 %). The study further revealed that in the intra-household roles in cocoa production, men and male children were more involved in the preparation of shade at the nursery (92.9 %), land clearing (100 % and 81.4 %), weeding 97 % and 81.4 %), spraying of herbicides (97.1 % and 75.7 %), spraying of pesticides (100.0 % and 78.6 %), removal of mistletoe (98.6 % and 81.4 %) and harvesting (100.0 % and 84.3 %), respectively. Women and female children were majorly involved in the sun-drying of cocoa beans (82.9 % and 90.0 %), while all the family members including men, women, male and female children were involved in nursery maintenance (92.9 %, 80 %, 78.8 %, and 74.3 %). The planting of improved seedlings and application of manure/fertilizer per unit area from more family labour (marriage or family size) increases yield. Ali et al. [52] noted that factors including household size, marital status, number of years working as a cocoa farmer (experience), support received from Non-Governmental Organizations (NGOs), farm size, and family labour significantly influence the extent of fertilizer adoption and application. Kanu [53] also reported that marital status, household size, and farm size, among others, influence the adoption of improved cocoa varieties. He further stated that 62 % of the cocoa farmers were married which means that they received assistance from their family members (wives, children, and relatives).

Although most farmers rated their cocoa yield as good and excellent, perhaps a reflection of the past decades, or to avoid negative confession, half of them indicated reduced production in the recent time. Age, occupation, household size, and marital status have a significant positive relationship with farmers' perception of cocoa yield while gender and education do not. Age is expected to be associated positively with experience, which is a factor of human perception Carstensen et al. [54]. Opinions on cocoa yield over the decades and the impact of climate will require some level of maturity from the farmers. Similarly, the farmers who have lived for some years are in a position to have more children or marry a second wife to provide the needed manpower on the cocoa farms. Going by the submission of Oyekale [4], the positive relationship between household size will significantly decrease technical inefficiency. On the other hand, the insignificant relationship between education level with farmers’ perception contradicts the finding of [54]. According to them, education is expected to influence the perception of farmers on climate change and enhance the adaptation of innovation. Generally, the basic education needed by farmers for engaging in cocoa farming is simple and often learned from older farmers which may not be connected with formal school training.

### 4.2 Farmers’ perception of climate in nexus with climate data on cocoa yield

Most farmers identified low sunshine, low rainfall, and low humidity as correlates of good cocoa yield. Also, the majority of the farmers were aware that high temperatures, high rainfall, humidity, wind speed, and sunshine period retard the yield of cocoa. While the farmers might not have the capability to quantify the parameters assessed, they will compare them to normal expected climate conditions during the different seasons. For instance, Obatolu et al. [55] noted that several factors including rainfall, temperature, sunlight, and humidity as well as soil nutrient status, pests, diseases, and farming practices have an interrelated impact on the growth of the cocoa plant. The temperature needed for optimum yield should not exceed 32 °C [56]. The projection by the Intergovernmental Panel on Climate Change (IPCC) [57] revealed that a slight increase in global temperatures (1–2 °C) will lead to about a 50 % decrease in agricultural production in developing countries. Temperature is important to speed up the photosynthetic rate and produces energy for warming the soil, plants, and the air, but when it is too high, a favourable condition is provided for capsids, a disease of cocoa [58]. Therefore, the provision of shade over cocoa trees becomes necessary as a control. However, the farm should not be overshaded as this could have a negative effect. Ruf and Schroth [59] reported that some farmers sometimes shade their farms to the point that new cocoa seedlings get spindly while searching for light, and tend to produce pods 2–3 m high thereby causing difficulty in harvesting and disease control.

Farmers complained that too much rainfall and increased relative humidity affect cocoa pod spraying effectiveness and cocoa yield. Confirming this, the below-average yield recorded in the study area from 1999 to 2004 corresponds to the period of above-normal rainfall. Lawal and Omonona [5] noted that high rainfall is detrimental to some metabolic processes that contribute to pod development and that an increase in relative humidity increases the incidences of the fungal disease known as ‘black pod disease’. Anim-Kwapong and Frimpong [16] also observed that black pod disease is a major threat to cocoa production when the relative humidity is very high. Too much rainfall also slows down the drying and processing of cocoa seeds, thereby reducing the value of the beans and an increase in the cost of processing [60]. However, the long period of cocoa failure that spans 6 years partly resulted from high temperatures and sunshine on one hand, and low rainfall and humidity on the other hand. Harsant et al. [61] reported that extreme temperatures and decreased rainfall led to an increase in potential crop evapotranspiration, an increased crop water requirement, additional water stress on crops, reduced pollen viability, embryo abortion, and ultimately reduced seed yield. This period is characterized by drought. Drought results in lower yield by reducing bean size and increasing the level of Capsid infestation [62]. High wind speed destroys cocoa flowers during the flowering period. Clay [63] reported that damage from heavy winds is a concern and cocoa trees should be protected by forest cover or windbreaks. Moreover, only a few of the farmers (10 %) identified that low wind speed had a positive influence on cocoa yield. This shows that most of the farmers focused more on the effect of heavy wind on cocoa yield than the benefits of low wind speed including pollination. The farmers noted that climate change results in changes in the timing of rainfall, growing seasons, low soil moisture content, and low cocoa yield. Cocoa farming contributes to global climate change through deforestation that disrupts local weather patterns and causes carbon emissions. High temperatures and droughts reduce cocoa planting. Farmers will not be able to grow much cocoa during this period and are forced to expand into new areas, thereby triggering the vicious cycle of further carbon emissions. Recent studies show that land suitable for cocoa production in West Africa will decrease significantly soon as a result of climate change. In some areas, longer or more intense dry seasons may adversely affect cocoa growing. Sofoluwe et al. [64] reported that a slight temperature change is likely to greatly affect cocoa yields and some types of cocoa plants are already at their temperature limit, causing a reduction in yields and potentially stunting growth. They further asserted that an increase in temperature decreases rainfall, thereby negatively impacting cocoa crop health and growth. The yield of some crops would decrease by a rise in global temperature of 0.2 °C per decade or 1 °C by 2040 with the smallest increase in the tropics. This would threaten global food security, with the most effect on developing nations [65].

It was observed that the answers of the most experienced farmers (above 60 years) showed that they had a better understanding of the effect of the weather parameters on cocoa yield than the least experienced (15–30 years). The most experienced farmers understood that high rainfall, high temperature, high wind speed, high sunshine, and high relative humidity hurt cocoa yield. For instance, the monthly rainfall record for forty-two years showed that rainfall is very high in the study area, especially during the wet season from March to October. Adejuwon [66] noted that 51 mm of rainfall is required for the flourishing of agricultural activities. This amount would guarantee that crops receive enough water for their survival. The range of 80 mm–205.5 mm rainfall is an indication of the availability of excess water that could cause dampness, hence harbour diseases especially black pod disease. This supports the view of the most experienced farmers that high rainfall harmed cocoa yield. The farmers associated high rainfall with isolated long-time heavy rainfall. This type of rainfall that mostly affects cocoa yield often occurs from June to October, particularly the rainfall peak period of ‘double maxima’ in July and September. Farmers often count their losses during this period of wet season as a result of the emergence of black pod disease that attacks cocoa pods due to high rainfall and humidity. Most of the farmers especially those that planted cocoa in the lowland areas often experience waterlogging and flooding during the period of heavy rainfall. At times, harvested cocoa pods are swept away by the flood. Farmers were aware that high wind speed results in mechanical damage to crops generally, and cocoa in particular, especially when cocoa plants were damaged by trees. They know that high sunshine and temperature are inimical to cocoa yield, and that is why they recognize the need to provide shade over cocoa plants. Intense sunshine and high temperatures become more hurtful during drought years and/or when dry spell is in a continuum or incessant. This category of farmers also noted that low temperature, low rainfall, low sunshine, and low wind speed had a positive effect. This reflection confirmed the adage that says ‘experience is the best teacher.’ Nevertheless, the view of some of them that low relative humidity had no effect, and the claim of a few others that high relative humidity had a positive influence on yield may be due to the insidious nature of the humidity effect, unlike rainfall. Furthermore, their assertion which appears different from scientific knowledge may be linked to their dependence on native knowledge and lack of formal education that is needed to associate high humidity with rainfall [67]. On the other hand, good education could have been responsible for the right answer provided by the ages of 31–45 years. However, farmers aged 15–30 years noted that high wind speed, low temperature, low rainfall, and low sunshine did not affect the cocoa yield. The reason why most farmers in this age group did not know the effect of these parameters on yield is linked to the lack of adequate experience in cocoa farming. Therefore, more education is needed for younger farmers.

The cocoa yield was dominated by the above-average yield from 2005 to 2019 except in 2006 while the rest were below-average yields. The above-average yield of crops could be attributed to favourable climatic conditions. An unprecedented rainfall deficit has been reported as a cause of a reduction in crops, particularly millet yield in Nigeria while the decrease of flower and pod production of bean plants by 50 % was attributed to soil water deficit [68,69]. Intergovernmental Panel on Climate Change (IPCC) [70] projected that some of these events will hurt agricultural production. Projected impacts vary across crops and regions, with about 10 % of projections for the 2030–2049 period showing yield gains of more than 10 %, and about 10 % of projections showing yield losses of more than 25 %, compared with the late 20th century. The multiple regression results revealed that meteorological parameters have no significant influence on cocoa yield. This implies that the yield of the crop is explainable by other factors than meteorological elements considered. Factors including the type of soil, soil fertility, insect and pest attack, and agronomy practices (choice of cultivars, planting density, and pesticides and herbicides) might influence the yield [13,71]. However, some of these factors were directly or indirectly affected by climate.

The major difficulties observed by the farmers with cocoa production include parasite infestation, weed management, and unfavourable weather condition in that order. They identified weather conditions as a less important problem to pests and weeds because they were unable to recognize that these factors are either directly or indirectly influenced by climate. They indicated government effort in the provision of incentives such as a loan, cocoa seedlings, and chemicals for pest control. The provision of incentives by governments and donor agencies has been identified as part of the needed intervention for increased agricultural production in Nigeria [72,73].

The suggested measures for improving cocoa production which included proper management practices, proper maintenance of the farm, and appropriate use of chemicals are indeed crucial for better management of the cocoa farm. David [74] and Azhar [75] reported that proper management practices, which include the use of disease-resistant planting materials, fertilizer programming, and integrated pest management, will minimize the negative impact on the environment, enhance soil fertility, and thus, lead to environmental sustainability. The cocoa farm can be properly maintained by removing and burning diseased and dead cocoa trees, removing dead trees and stumps from the farm, replacing seedlings that have not grown, removing weeds and keeping the soil covered, pruning the cocoa trees, and protecting the cocoa trees from insects and diseases. Also, planting trees will: protect the young cocoa plants from too much radiation, wind, and heavy rain; decrease overall temperature and temperature extremes; reduce light quantity and intensity which prevents excessive vegetative growth; reduce the rate of decomposition of soil organic material (due to reduced soil temperatures); suppress weed growth; lower the risk of some pests and diseases and thus lowering the need for agrochemicals; keep the soil moisture and provide carbon sinks and release oxygen that replenishes fauna in the surroundings [76,77].

## 5 Conclusion

The study has shown that cocoa farmers are aware of the declining state of cocoa production in the study area. They demonstrated the fact that climate parameters affect the production of cocoa both positively and negatively depending on the swing from low to high in normal climate conditions. The study has also shown that the most experienced farmers had a better understanding of the effect of weather parameters on yield than the least experienced. The interaction of various climatic elements with cocoa yield revealed that both climatic and non-climatic factors influence the yield of cocoa in the study area.

The consequences of high temperature, rainfall, humidity, wind speed, and sunshine include reduced cocoa yield, an increased infestation of pests and diseases, mechanical damage to the cocoa plant, and weed growth. Since climate directly or indirectly affects other factors influencing cocoa yield, the solution lies in the proper monitoring of these climatic parameters. The measures for improving cocoa production will need to be addressed more scientifically by the Government agencies through the Agriculture Extension Officer in the cocoa-producing areas. Finally, cocoa farmers need capacity building in respect of their farms’ management because of the climate variability and the impending climate change impacts.

## Declarations

.

## 6 Data availability statement

Data has been deposited into a publicly available repository. It was attached as part of the submission when it was demanded by the Journal before review. I do not know the name of the repository and the accession number.

## Funding statement

This research did not receive any specific grant from funding agencies in the public, commercial, or not-for-profit sectors.

## CRediT authorship contribution statement

**Joseph O. Adejuwon:** Writing – review & editing, Visualization, Supervision, Project administration, Methodology, Investigation, Formal analysis, Data curation, Conceptualization. **K.E. Tewogbade:** Writing – original draft, Visualization, Investigation, Funding acquisition, Data curation, Conceptualization. **O. Oguntoke:** Writing – review & editing, Validation, Supervision. **G.C. Ufoegbune:** Writing – review & editing, Supervision.

## Declaration of competing interest

The author declares that there is no conflict of interest regarding the publication of the article titled ‘Comparing farmers’ perception of climate effect on cocoa yield with climate data in the Humid Zone of Nigeria’
