# Supplementary material for: Comparing farmers’ perception of climate effect on cocoa yield with climate data in the Humid zone of Nigeria
Source: Heliyon. 2023 Dec 3;9(12):e23155. doi: 10.1016/j.heliyon.2023.e23155 (PMC10746452; doi:10.1016/j.heliyon.2023.e23155)
Supplement: Multimedia component 1 [file mmc1.doc]

**AN INVESTIGATION OF METEOROLOGICAL PARAMETERS AS CONSTRAINING FACTORS ON COCOA YEILD IN OSUN STATE.**

**QUESTIONNAIRE**

Dear Respondent,

This questionnaire is design to examine the influence of meteorological parameters on cocoa yield in Osun state.

Kindly provide honest and reliable answers to the items contained in this questionnaire.

Please take note that all information supplied shall be treated with utmost confidentiality.

Thank you sincerely for your anticipated cooperation.

**SECTION A BIO-DATA**

1. Name: ………………………………………….( Optional)

2. Name of your area/Local Government Area ………………………………………

3. Gender: Male Female

4. Age: (a) 15-30 years (b) 31-45 years (c) 45-60 years

(d) Above 60

5. Occupation: (a) Farmers (b) Civil servant (c) Trader (d) Artisan

6. Household size: (a 1 (b) 2-3 (c) 4-6 (d) Above 6 members

7. Marital status (a) Single (b) Married (c) Divorce

8. Are you educated: Yes No

SECTION B. Farmer’s perception on cocoa yield/ production.

9. How do you rate the yield of cocoa in your Local government? Good Poor Excellent Fair

10. In terms of income, is coca yield more income for your local government? Yes No

11. Is cocoa production on a large scale level? Yes No

12. How many tonnes of cocoa do you produce yearly? ………………..

13. Has the government provided assistance to cocoa farmers recently? Yes No

If yes, in what form (1) Pesticide (2) Herbicides (3) Fertilizers (4) Loans

14. In recent years as the yield of cocoa reduce? Yes No

15. What are the likely chemical used in the cultivation of cocoa? ....................

16. Which season of the year is best for cocoa production? Dry Wet

17. What is the specie of cocoa planted on your farm? Hybrid Traditional

18. What is the best farming method for cocoa cultivation? ………………

19. What are the dangers associated to cocoa yield?

……………………………………………………………………………………………………………………………………………………………………………………………………………………………………………………………………………………………………………………....

20. What is the effort of the government in increasing cocoa yield?

21. What are the likely things that can improve the productivity of cocoa?

……………………………………………………………………………………………………

22. How do you get your seed for cocoa cultivation?

23. Do you have any drought tolerant cocoa specie? Yes No

24. Can drought tolerant cocoa specie grow well in Osun state/ LGA? Yes No

25. Is your farm size large? Yes No

26. How many cocoa farms do you have? (a)1 (b)2 (c) 3 (d) Above 4

27. What are thr input used in cocoa cultivation? …………………

28. What are the implement used in the cultivation of cocoa? Hoe Cutlass Mechanized Others

29. What is the cost of labour in cocoa production? (a) 100000 (b) 100000-200000 (c) 200000- 250000 (d) 250000-300000

C: Farmers perception on the influence of weather on cocoa yield.

How is cocoa yield affected by the following?

| Weather parameters | Positive | negative | No effect | Not sure |
| --- | --- | --- | --- | --- |
| High temperature |  |  |  |  |
| Low temperature |  |  |  |  |
| Low rainfall |  |  |  |  |
| High rainfall |  |  |  |  |
| High humidity |  |  |  |  |
| Low humidity |  |  |  |  |
| High wind speed |  |  |  |  |
| Low wind speed |  |  |  |  |
| Weather condition |  |  |  |  |
| High sunlight radiation |  |  |  |  |
| Low sunlight radiation |  |  |  |  |

30. Name the likely disease that affects cocoa production in your area?

(1) ……………………………….. (2) ………………………………… (3) ……………..

(4) ………………………………..

31. In what year is worst cocoa yield recorded in your area? …………………….

32. What are the varieties of your cocoa? ………………………….

33. What variety do you plant? ......................................................

34. What is the reason behind that variety? ……………………………………………
